# Supplementary material for: 3D reconstruction of bird flight trajectories using a single video camera
Source: PLoS One. 2022 Aug 24;17(8):e0271618. doi: 10.1371/journal.pone.0271618 (PMC9401184; doi:10.1371/journal.pone.0271618)
Supplement: S1 File — (DOCX) [file pone.0271618.s002.docx]

**Supplementary Information**

**3D reconstruction of bird flight TRAJECTORIES**

**using a single video camera**

M.V. Srinivasan, H.D. Vo and I. Schiffner

**SECTION A**

**Experimental subjects**

The birds used in this study were drawn from a group of 6 mature English Budgerigars (*Melopsittacus undulatus*). They were purchased from a local pet shop at the age of approximately one month and were housed in an outdoor aviary measuring 4 m in length, 2 m in width and 2.2 m in height. The mesh walls of the aviary provided a natural diurnal light cycle. Perches were placed in areas with regular sunlight as well as in more sheltered areas shielded from inclement weather. The aviary included ample natural foliage, as well as activity toys such as ladders and swings. Birds were moved (individually, or in pairs) from the aviary to the experimental arena in 47cm x 34.5cm x82cm cages, to conduct experiments about two to three times a week. Humane endpoints for the use of an animal were (a) signs of fatigue and/or (b) reluctance to fly. However, these endpoints were never reached during this study.

**Training**

The experimental arena was a chamber of dimensions 7.28 m (length) x 1.36 m (width) x 2.44 m (height). The birds were initially trained to take off from a perch at one end of the chamber (tunnel) and fly through a vertically oriented aperture to reach the other end of the chamber for no more than five times per day, before their flights were recorded. The take-off perch was either held by an experimenter or was affixed to the wall. A bird cage, 50 cm high, containing familiar birds was placed on the floor at the other end of the chamber. This motivated the experimental bird to take off from its perch, fly through the aperture and land on the birdcage. After training for one day, the bird cage was replaced by a second perch on which the trained birds landed. In order to minimize effects of fatigue or stress on the birds’ flight performance, an individual bird was never flown more than 10 times on any given day. The laboratory was in regular close contact with a professional veterinary clinic for health checks and advice on any emergency issues.

Two randomly selected birds were used in this study. There was no basis for choice or exclusion of individuals. Bird Four (male, 9 years old, 58 g, 29 cm wingspan) provided the results described in the main text, and bird Nemo (male, 9 years old, 64 g, 33 cm wingspan) the results described in the SI. The study does not require a large sample size, because its goal is only to develop and describe a methodology for tracking and reconstructing bird flight trajectories in three dimensions. The animal species, strain and gender are not relevant to the findings or conclusions of this study. The accuracy of the methodology is evaluated by comparing the results that it delivers (computed 3D locations of the bird) with ground-truth information from a model bird positioned at known 3D locations. Analyses of the method’s accuracy are provided in Fig 13 of the main text, and in Table S1 of the Supplementary Information.

**SECTION B**

***Table S1***

*Test of accuracy of 3D head position measurements. X, Y and H represent (in cm) the true co-ordinates of a test target along the axial (length), width and height of the tunnel, respectively. X calc, Y calc and H calc are the calculated values of these co-ordinates, and X error, Y error and H error represent the respective errors. The standard deviation of the errors (SD) are given at the end of the table. The 5 missing measurements pertain to target positions whose floor projections fell outside the grid.*

| \| **Points** \| **X (cm)** \| **X calc** \| **X error** \| **Y (cm)** \| **Y calc** \| **Y error** \| **H (cm)** \| **H calc** \| **H error** \| \| --- \| --- \| --- \| --- \| --- \| --- \| --- \| --- \| --- \| --- \| \| 1 \| 36.75 \| - \| - \| 115.5 \| - \| - \| 91 \| - \| - \| \| 2 \| 36.75 \| - \| - \| 75.5 \| - \| - \| 91 \| - \| - \| \| 3 \| 36.75 \| - \| - \| 35.5 \| - \| - \| 91 \| - \| - \| \| 4 \| 116.75 \| 114.59 \| -2.16 \| 115.5 \| 115.12 \| -0.38 \| 91 \| 89.24 \| -1.76 \| \| 5 \| 116.75 \| 116.25 \| -0.50 \| 75.5 \| 75.25 \| -0.25 \| 91 \| 92.21 \| 1.21 \| \| 6 \| 116.75 \| 112.91 \| -3.84 \| 35.5 \| 35.43 \| -0.07 \| 91 \| 87.53 \| -3.47 \| \| 7 \| 216.75 \| 216.47 \| -0.28 \| 115.5 \| 115.46 \| -0.04 \| 91 \| 87.86 \| -3.14 \| \| 8 \| 216.75 \| 216.61 \| -0.14 \| 75.5 \| 75.65 \| 0.15 \| 91 \| 95.20 \| 4.20 \| \| 9 \| 216.75 \| 216.49 \| -0.26 \| 35.5 \| 35.94 \| 0.44 \| 91 \| 89.37 \| -1.63 \| \| 10 \| 316.75 \| 316.37 \| -0.38 \| 115.5 \| 115.33 \| -0.17 \| 91 \| 90.34 \| -0.66 \| \| 11 \| 316.75 \| 316.76 \| 0.01 \| 75.5 \| 75.45 \| -0.05 \| 91 \| 91.88 \| 0.88 \| \| 12 \| 316.75 \| 318.51 \| 1.76 \| 35.5 \| 34.72 \| -0.78 \| 91 \| 87.28 \| -3.72 \| \| 13 \| 36.75 \| - \| - \| 115.5 \| - \| - \| 79 \| - \| - \| \| 14 \| 36.75 \| 40.32 \| 3.57 \| 75.5 \| 75.69 \| 0.19 \| 79 \| 80.02 \| 1.02 \| \| 15 \| 36.75 \| - \| - \| 35.5 \| - \| - \| 79 \| - \| - \| \| 16 \| 136.75 \| 136.58 \| -0.17 \| 115.5 \| 115.62 \| 0.12 \| 79 \| 79.13 \| 0.13 \| \| 17 \| 136.75 \| 138.51 \| 1.76 \| 75.5 \| 75.51 \| 0.01 \| 79 \| 83.09 \| 4.09 \| \| 18 \| 136.75 \| 136.89 \| 0.14 \| 35.5 \| 36.15 \| 0.65 \| 79 \| 80.10 \| 1.10 \| \| 19 \| 236.75 \| 234.68 \| -2.07 \| 115.5 \| 116.37 \| 0.87 \| 79 \| 74.71 \| -4.30 \| \| 20 \| 236.75 \| 233.38 \| -3.37 \| 75.5 \| 76.13 \| 0.63 \| 79 \| 81.48 \| 2.48 \| \| 21 \| 236.75 \| 235.00 \| -1.75 \| 35.5 \| 36.42 \| 0.92 \| 79 \| 78.82 \| -0.18 \| \| 22 \| 336.75 \| 336.80 \| 0.05 \| 115.5 \| 114.59 \| -0.91 \| 79 \| 79.99 \| 0.99 \| \| 23 \| 336.75 \| 333.92 \| -2.83 \| 75.5 \| 75.25 \| -0.25 \| 79 \| 82.73 \| 3.73 \| \| 24 \| 336.75 \| 336.71 \| -0.04 \| 35.5 \| 35.94 \| 0.44 \| 79 \| 79.15 \| 0.15 \| \| 25 \| 36.75 \| 40.70 \| 3.95 \| 115.5 \| 114.27 \| -1.23 \| 65.5 \| 68.99 \| 3.49 \| \| 26 \| 36.75 \| 39.67 \| 2.92 \| 75.5 \| 75.91 \| 0.41 \| 65.5 \| 68.13 \| 2.63 \| \| 27 \| 36.75 \| 38.57 \| 1.82 \| 35.5 \| 35.85 \| 0.35 \| 65.5 \| 67.39 \| 1.89 \| \| 28 \| 136.75 \| 33.08 \| -3.67 \| 115.5 \| 114.09 \| -1.41 \| 65.5 \| 61.67 \| -3.83 \| \| 29 \| 136.75 \| 138.08 \| 1.33 \| 75.5 \| 74.88 \| -0.62 \| 65.5 \| 69.27 \| 3.77 \| \| 30 \| 136.75 \| 138.12 \| 1.37 \| 35.5 \| 35.90 \| 0.40 \| 65.5 \| 67.35 \| 1.85 \| \| 31 \| 236.75 \| 138.34 \| 1.59 \| 115.5 \| 115.72 \| 0.22 \| 65.5 \| 66.66 \| 1.16 \| \| 32 \| 236.75 \| 136.17 \| -0.58 \| 75.5 \| 75.56 \| 0.06 \| 65.5 \| 63.34 \| -2.16 \| \| 33 \| 236.75 \| 235.53 \| -1.22 \| 35.5 \| 34.93 \| -0.57 \| 65.5 \| 64.44 \| -1.06 \| \| 34 \| 336.75 \| 234.92 \| -1.83 \| 115.5 \| 115.93 \| 0.43 \| 65.5 \| 67.65 \| 2.15 \| \| 35 \| 336.75 \| 235.61 \| -1.14 \| 75.5 \| 76.21 \| 0.71 \| 65.5 \| 66.36 \| 0.86 \| \| 36 \| 336.75 \| 236.73 \| -0.02 \| 35.5 \| 36.80 \| 1.30 \| 65.5 \| 61.50 \| -4.00 \| \| 37 \| 396.75 \| 338.17 \| 1.42 \| 115.5 \| 115.09 \| -0.41 \| 65.5 \| 64.72 \| -0.78 \| \| 38 \| 396.75 \| 336.05 \| -0.70 \| 75.5 \| 75.66 \| 0.16 \| 65.5 \| 67.88 \| 2.38 \| \| 39 \| 396.75 \| 338.22 \| 1.47 \| 35.5 \| 36.40 \| 0.90 \| 65.5 \| 65.55 \| 0.05 \| \| 40 \| 36.75 \| 340.04 \| 3.29 \| 19.5 \| 18.79 \| -0.71 \| 65.5 \| 61.62 \| -3.88 \| \| 41 \| 136.75 \| 392.44 \| -4.31 \| 19.5 \| 18.66 \| -0.84 \| 65.5 \| 70.01 \| 4.51 \| \| 42 \| 236.75 \| 400.11 \| 3.36 \| 19.5 \| 19.27 \| -0.23 \| 65.5 \| 62.98 \| -2.52 \| \| 43 \| 336.75 \| 396.52 \| -0.23 \| 23.5 \| 23.48 \| -0.02 \| 65.5 \| 65.51 \| 0.01 \| \| 44 \| 396.75 \| 395.72 \| -1.03 \| 23.5 \| 23.11 \| -0.39 \| 65.5 \| 66.04 \| 0.54 \| \| **Averaged Error (cm)** \| \| \| **-0.07** \|  \|  \| **0.00** \|  \|  \| **0.21** \| \| **SD Error (cm)** \| \| \| **2.09** \|  \|  \| **0.61** \|  \|  \| **2.57** \| |
| --- | --- | --- | --- | --- | --- | --- | --- | --- | --- | --- | --- | --- | --- | --- | --- | --- | --- | --- | --- | --- | --- | --- | --- | --- | --- | --- | --- | --- | --- | --- | --- | --- | --- | --- | --- | --- | --- | --- | --- | --- | --- | --- | --- | --- | --- | --- | --- | --- | --- | --- | --- | --- | --- | --- | --- | --- | --- | --- | --- | --- | --- | --- | --- | --- | --- | --- | --- | --- | --- | --- | --- | --- | --- | --- | --- | --- | --- | --- | --- | --- | --- | --- | --- | --- | --- | --- | --- | --- | --- | --- | --- | --- | --- | --- | --- | --- | --- | --- | --- | --- | --- | --- | --- | --- | --- | --- | --- | --- | --- | --- | --- | --- | --- | --- | --- | --- | --- | --- | --- | --- | --- | --- | --- | --- | --- | --- | --- | --- | --- | --- | --- | --- | --- | --- | --- | --- | --- | --- | --- | --- | --- | --- | --- | --- | --- | --- | --- | --- | --- | --- | --- | --- | --- | --- | --- | --- | --- | --- | --- | --- | --- | --- | --- | --- | --- | --- | --- | --- | --- | --- | --- | --- | --- | --- | --- | --- | --- | --- | --- | --- | --- | --- | --- | --- | --- | --- | --- | --- | --- | --- | --- | --- | --- | --- | --- | --- | --- | --- | --- | --- | --- | --- | --- | --- | --- | --- | --- | --- | --- | --- | --- | --- | --- | --- | --- | --- | --- | --- | --- | --- | --- | --- | --- | --- | --- | --- | --- | --- | --- | --- | --- | --- | --- | --- | --- | --- | --- | --- | --- | --- | --- | --- | --- | --- | --- | --- | --- | --- | --- | --- | --- | --- | --- | --- | --- | --- | --- | --- | --- | --- | --- | --- | --- | --- | --- | --- | --- | --- | --- | --- | --- | --- | --- | --- | --- | --- | --- | --- | --- | --- | --- | --- | --- | --- | --- | --- | --- | --- | --- | --- | --- | --- | --- | --- | --- | --- | --- | --- | --- | --- | --- | --- | --- | --- | --- | --- | --- | --- | --- | --- | --- | --- | --- | --- | --- | --- | --- | --- | --- | --- | --- | --- | --- | --- | --- | --- | --- | --- | --- | --- | --- | --- | --- | --- | --- | --- | --- | --- | --- | --- | --- | --- | --- | --- | --- | --- | --- | --- | --- | --- | --- | --- | --- | --- | --- | --- | --- | --- | --- | --- | --- | --- | --- | --- | --- | --- | --- | --- | --- | --- | --- | --- | --- | --- | --- | --- | --- | --- | --- | --- | --- | --- | --- | --- | --- | --- | --- | --- | --- | --- | --- | --- | --- | --- | --- | --- | --- | --- | --- | --- | --- | --- | --- | --- | --- | --- | --- | --- | --- | --- | --- | --- | --- | --- | --- | --- | --- | --- | --- | --- | --- | --- | --- | --- | --- | --- | --- | --- | --- | --- | --- | --- | --- | --- | --- | --- | --- | --- | --- | --- | --- | --- | --- | --- | --- | --- | --- | --- | --- | --- | --- | --- | --- | --- | --- | --- | --- | --- | --- | --- | --- | --- | --- | --- | --- | --- | --- | --- | --- | --- |

**Results for bird Nemo**


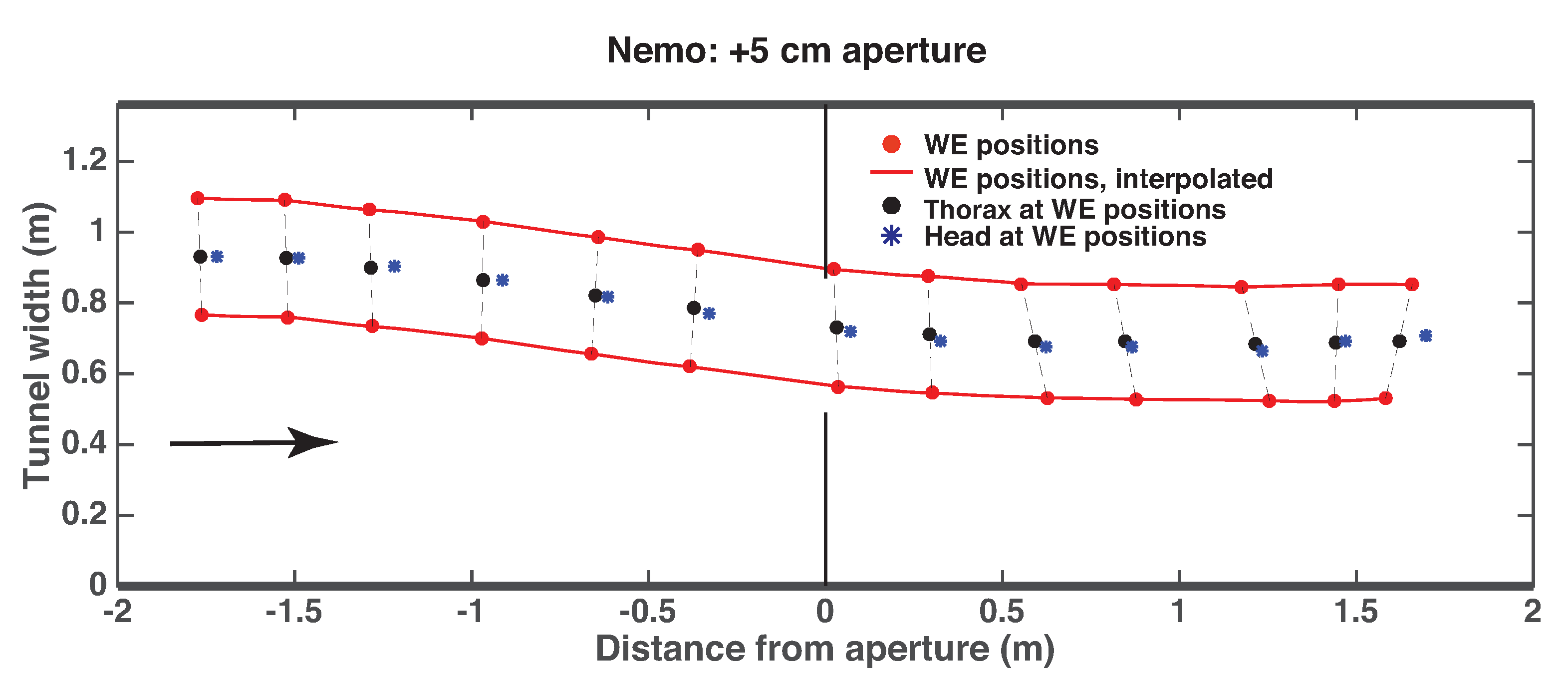


***Figure S1*** *Plan view of a reconstructed flight of bird Nemo. In this example the wingspan of the bird (Nemo) is 33 cm and it flies through a 38 cm aperture, which is 5 cm wider than the wingspan. Details are as in Figure 5.*

**
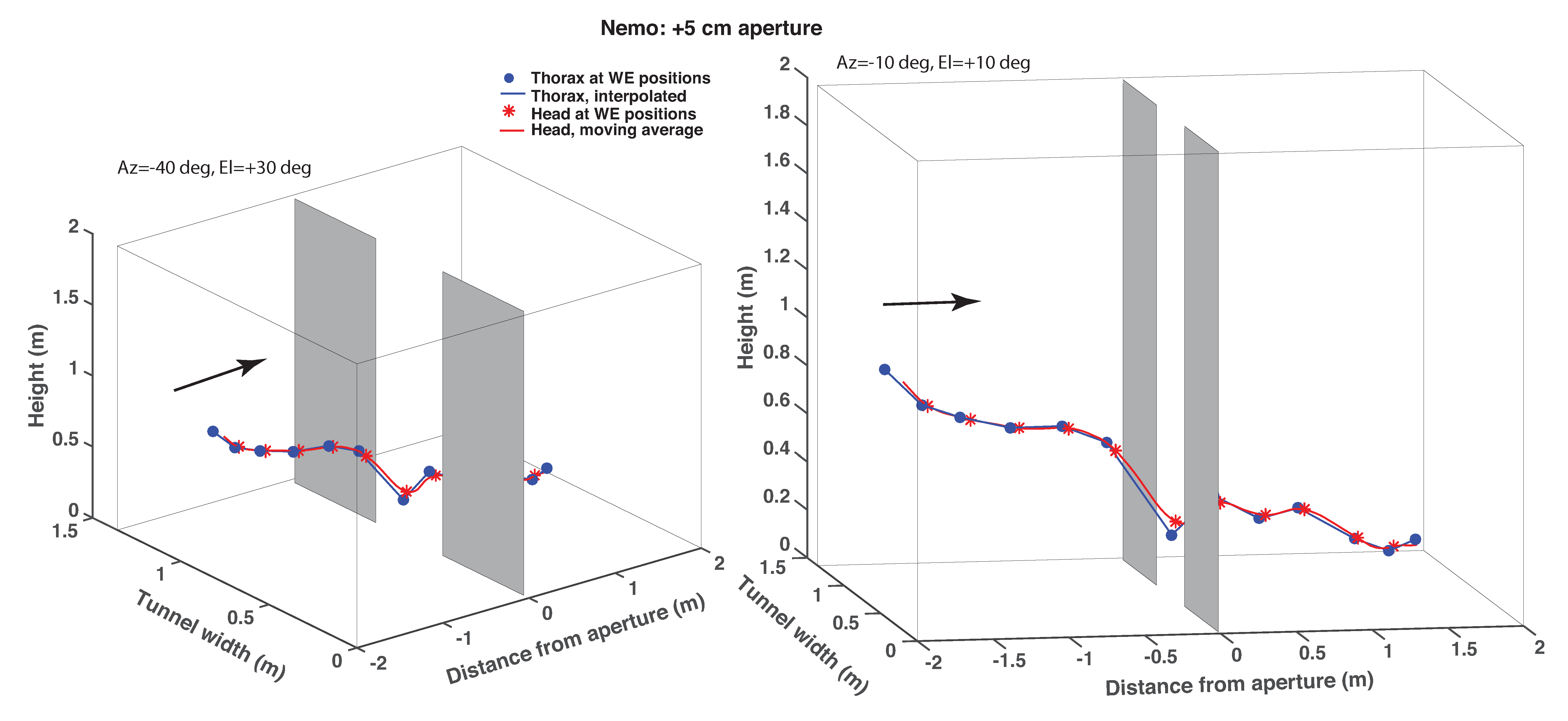
**

***Figure S2*** *Two 3D views of the trajectory shown in Figure S1, in which Nemo flies through an aperture that is 5 cm wider than its wingspan. Details are as in Figure 6.*


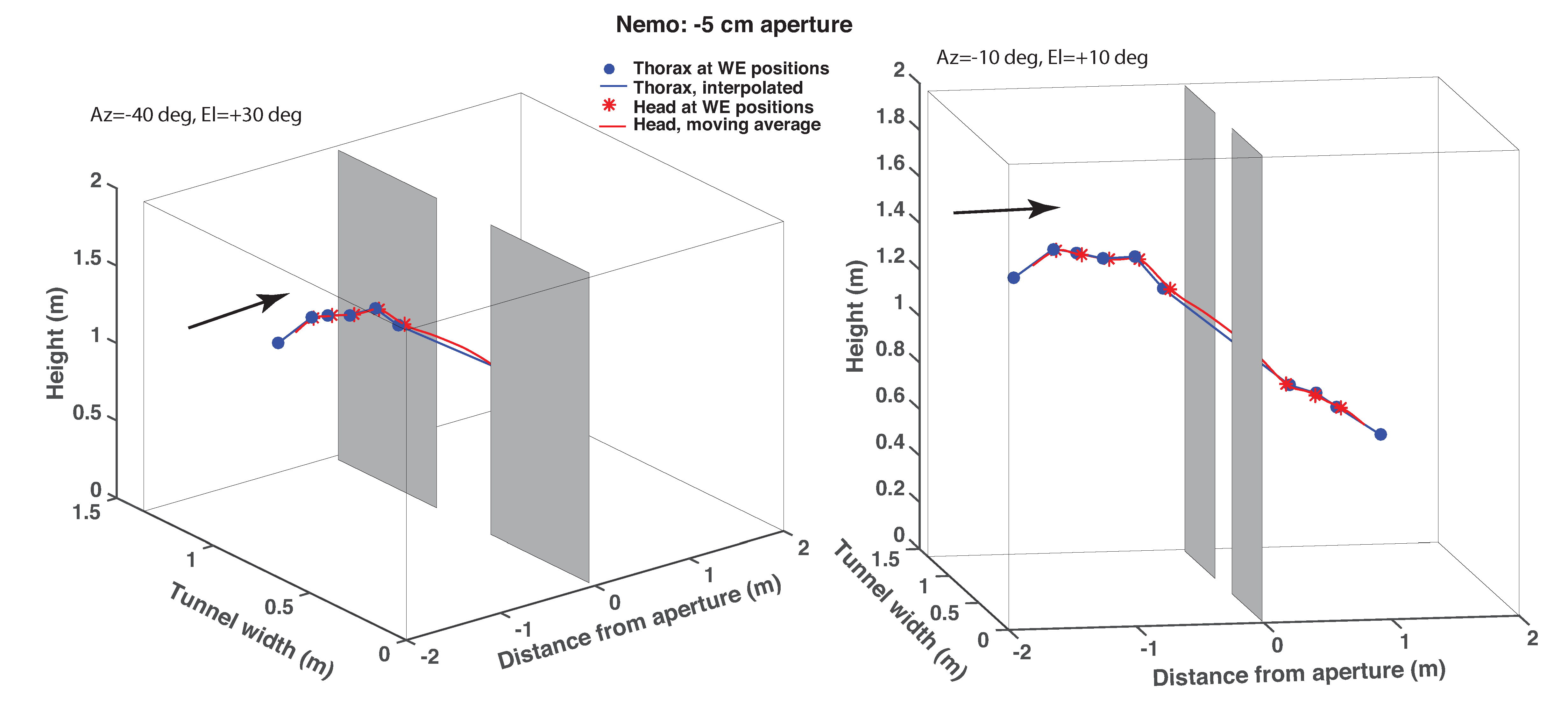


***Figure S3*** *Two 3D views of a trajectory of bird Nemo during flight through an aperture that is 5 cm narrower than its wingspan. Details are as in Figure 6.*


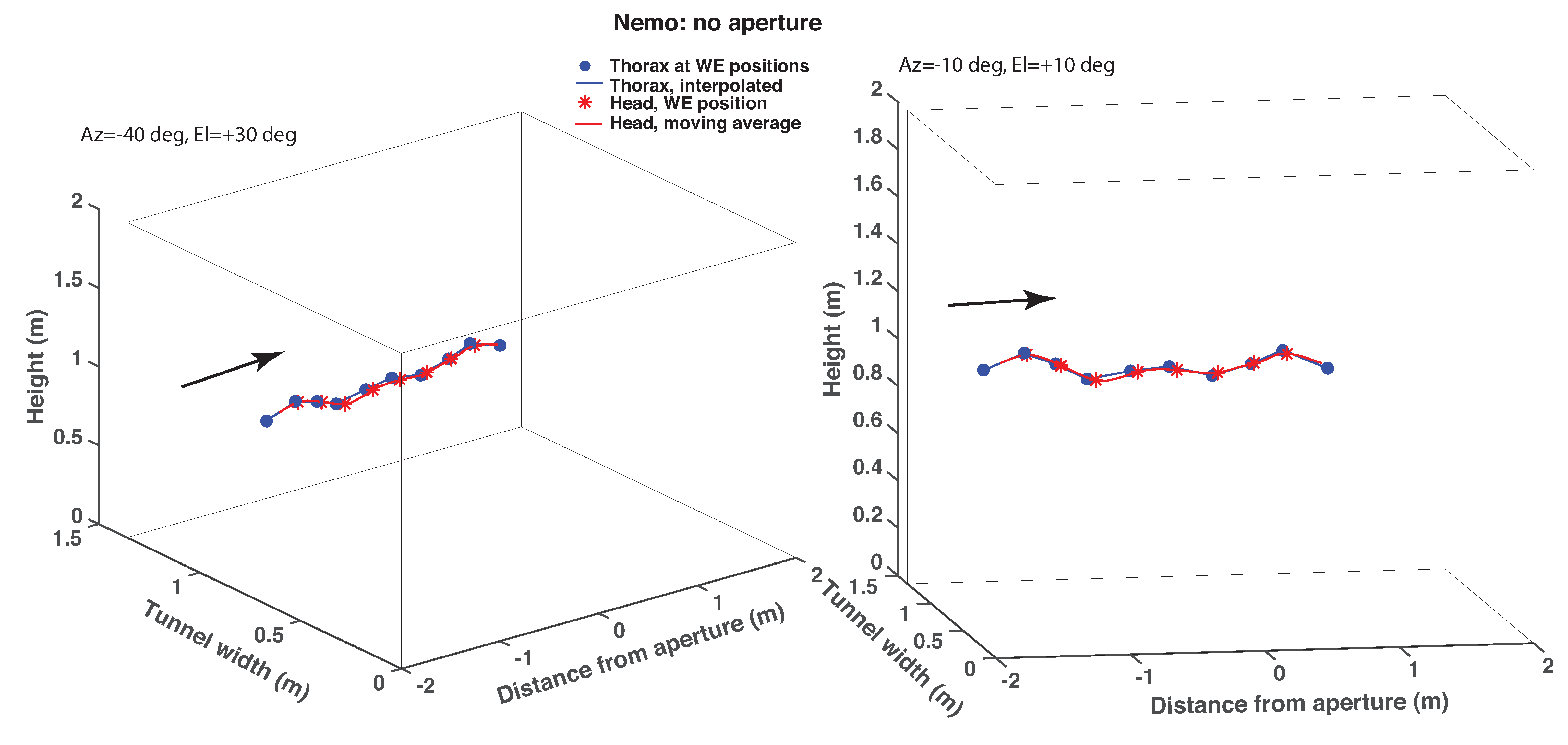


***Figure S4*** *Two 3D views of a trajectory of bird Nemo during flight through a tunnel which carries no aperture. Details are as in Figure 6.*


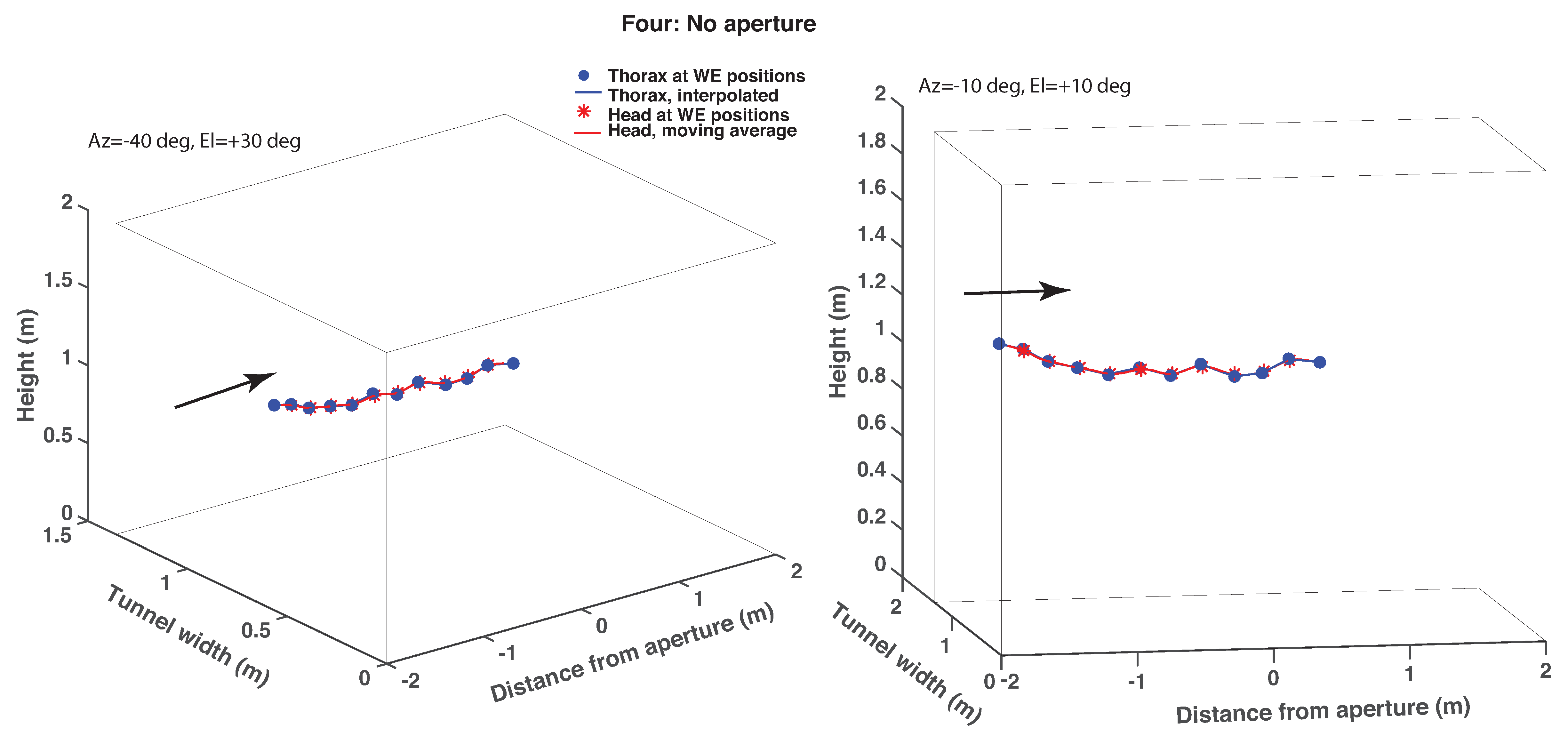


***Figure S5*** *Two 3D views of a trajectory of bird Four during flight through a tunnel which carries no aperture. Details are as in Figure 6.*


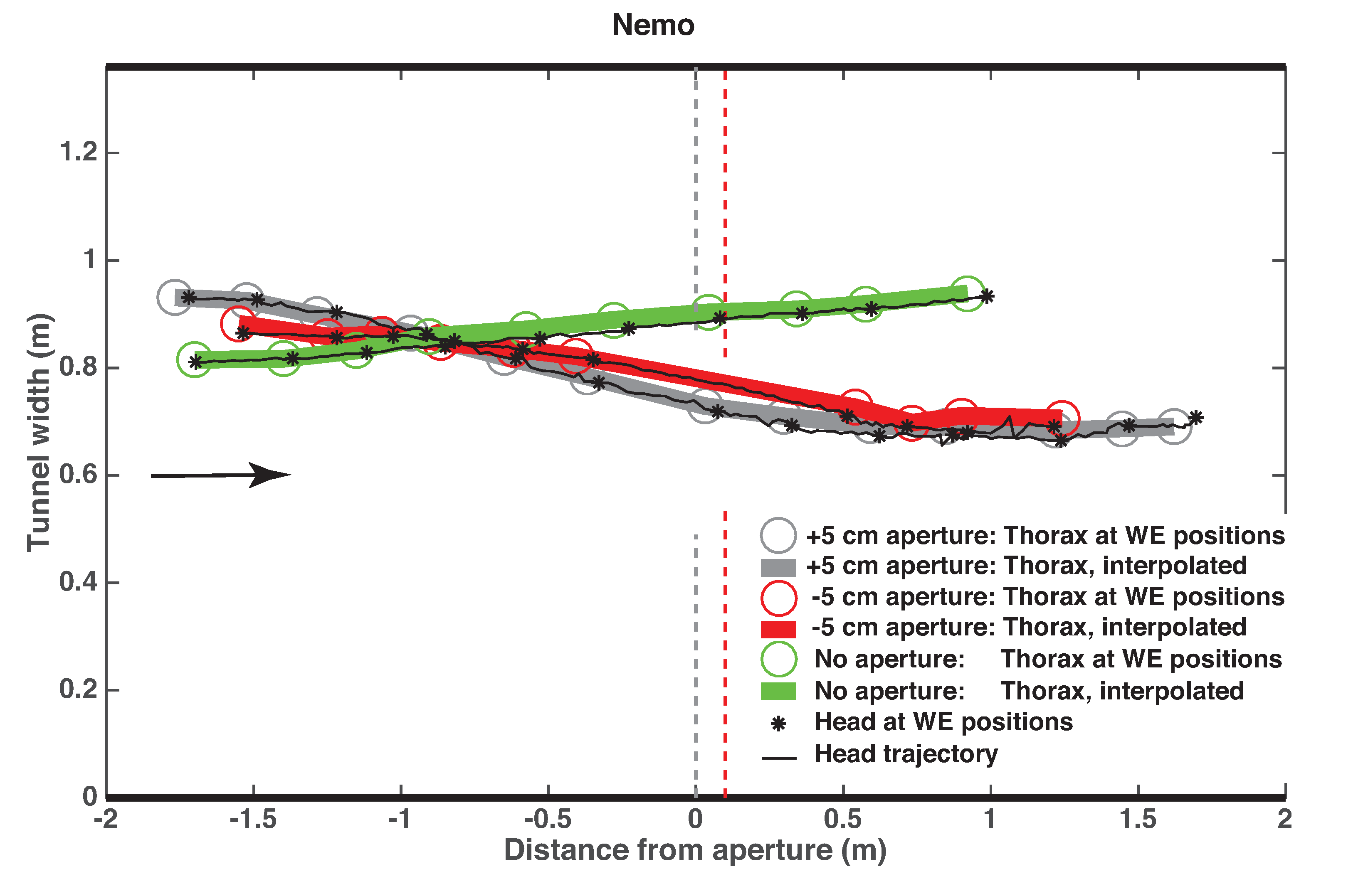


***Figure S6*** *Plan views of the reconstructed 3D trajectories for the narrow aperture condition (red), the wide aperture condition (grey) and the no aperture condition (green), for bird Nemo. Details are as in Figure 8.*

**
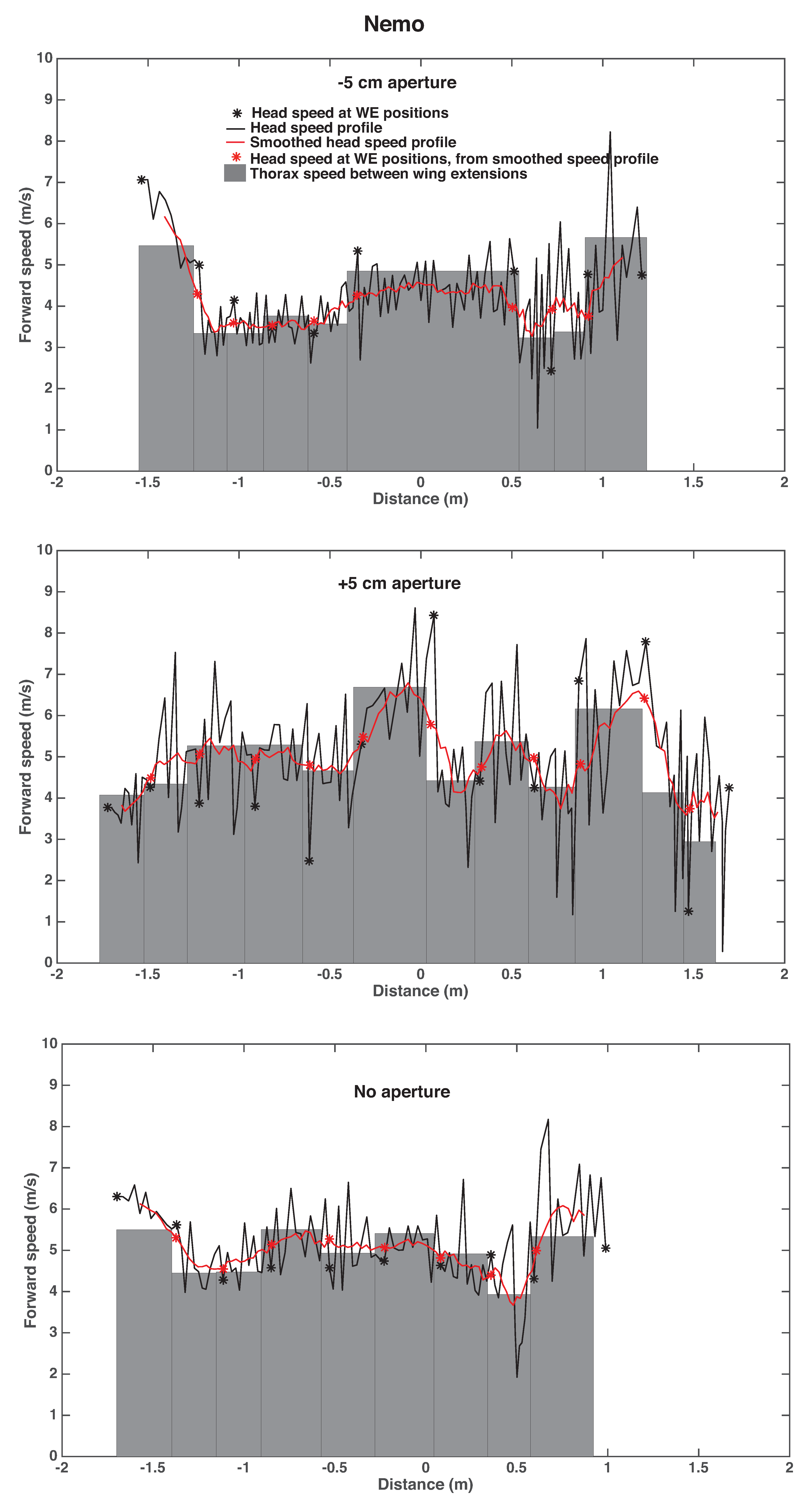
**

***Figure S7*** *Forward speed profile of bird Nemo during flight through the narrow aperture (top panel), the wide aperture (middle panel), and the empty tunnel (bottom panel). Details are as in Figure 9.*

**Supplementary Videos**

Two examples of the flights of bird *Four*, as captured by the overhead video camera, are shown in videos SV1 and SV2. In video SV1 the bird passes through an aperture that is 5 cm wider than its wingspan (+5 cm) without closing its wings. In SV2 the bird passes through an aperture that is 5cm narrower than its wingspan (-5 cm), and closes its wings before entering the aperture. The calibration grid is not visible in these videos because its construction and camera calibration were carried out after the flights were filmed.

SV1: <https://drive.google.com/file/d/1kKwyJ8IJtk3q7357YKiwodz-Ydlmya0M/view?usp=sharing>

SV2: <https://drive.google.com/file/d/1n3FjqKH_oMk5Wfsb_xqQQS7zVJ9BhCkd/view?usp=sharing>

**SECTION C**

**Derivation of extended calculation**

Figure S8 illustrates the general case in which a bird, at an arbitrary 3D position in the arena, is filmed by the overhead camera. The floor caries a calibration grid (not shown). The bird is shown rolling to the left (the left wingtip is lower than the right). We wish to determine the height (h) of the bird above the floor, and the roll angle (α). R and L denote the positions of the right and left wingtips when the wings are fully extended; O denotes the thorax point, as defined in the main text. X, Y and Z denote the projections of the images of R, L and O on the floor. These are locations determined by grid interpolation, as described in Section 2.1 of the main text. U, the intersection of the camera axis on the floor, is the origin of the 3D coordinate system with coordinates (0,0,0). Since X,Y and Z are on the floor, their z-coordinates are zero. Other points and geometrical variables are defined in the figure.


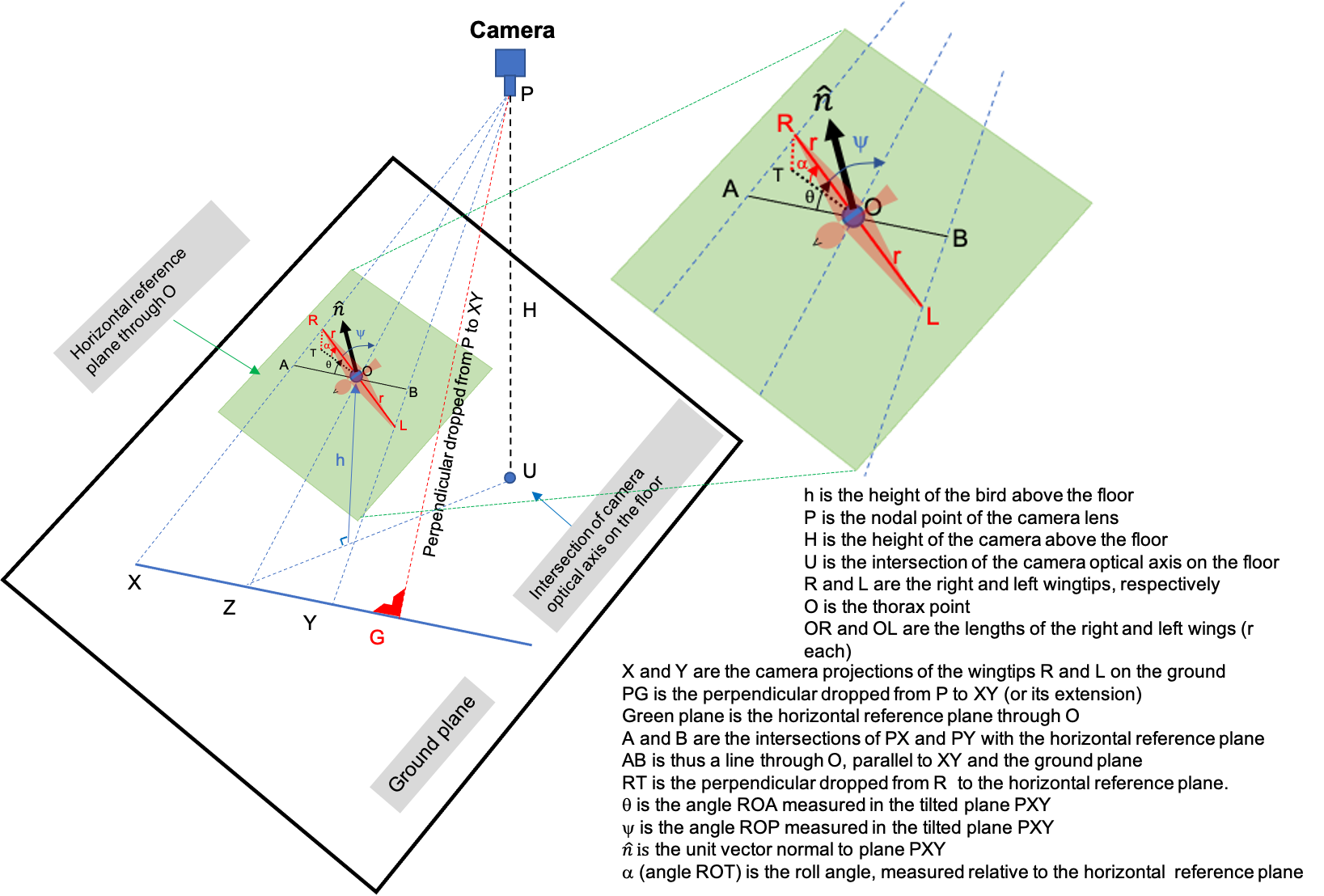


***Figure S8*** *Geometry pertaining to calculation of the height and the roll angle of a bird at an arbitrary 3D location.*

Figure S9 shows a perpendicular view of the plane PXY, including definitions of additional variables (including the angles φ1, φ2) that are relevant to the calculation.


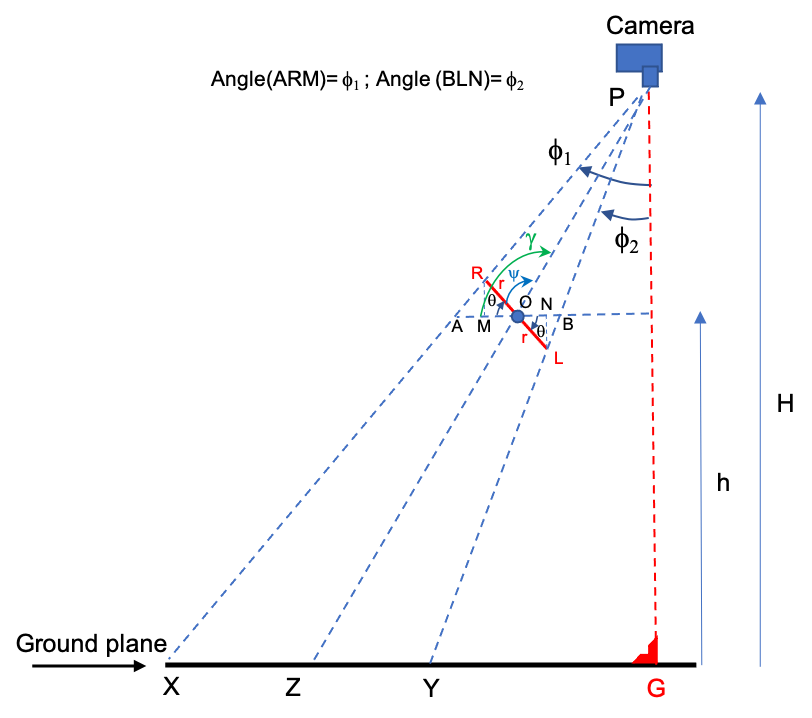


***Figure S9*** *Perpendicular view of the plane PXY in Figure S8. Details in text.*

The calculation is performed in three stages. First, we determine θ, which is the angle ROA measured in the plane PXY (See Figures S8, S9; Note that this angle is different from the roll angle α). Next, we use the calculated value of θ to determine the height (*h*) of the bird (this is the height above the floor of the thorax point O). Finally, we use the values of θ and *h* to calculate the roll angle α, which is the angle ROT in Fig S8.

***Stage 1: Calculation of θ***

Since X, Y and Z are the camera-projected points of the right and left wingtips (R,L) and the thorax point (O) on the floor, their floor coordinates, and hence the distances [XY], [XZ] and [YZ] can be determined.

Next, we determine the location of the point G, which is the intersection of the perpendicular dropped from P to the line connecting X and Y (or its extension). The location of G relative to X and Y can be determined by calculating the 3D vectors $\bar{PX}$, $\bar{PY}$ and $\bar{XY}$, and the unit vector in the direction of $\bar{XY}$, which we denote by $\bar{XY}_{u}$. The length [GX] is the projection of $\bar{PX}$ on $\bar{XY}$, which is given by $\bar{PX}.\bar{XY}_{u}$ where (.) denotes the vector dot product. Similarly, the length [GY] is given by $\bar{PY}.\bar{XY}_{u}$.

From the geometry of Figure S9, it is clear that angle(ARM) = φ1, and angle(BLN) = φ2.

Therefore,

$\tan\emptyset_{1}=\frac{[AM]}{[RM]}=\frac{[GX]}{H}$ (S1)

and

$\tan\emptyset_{2}=\frac{[BN]}{[LN]}=\frac{[GY]}{H}$ (S2)

Since [GX], [GY] and H are known, the angles φ1 and φ2 can be evaluated from (S1) and (S2).

From Figure S9, we have

$\left[ OM \right]=\left[ ON \right]=r\cos\theta$; $\left[ AM \right]=r\sin\theta\tan\phi_{1}$; and $\left[ BN \right]=r\sin\theta\tan\phi_{2}$

We can thus write

$\frac{[OA]}{[XZ]}=\frac{\left[ OM \right]+[AM]}{[XZ]}=\frac{r\cos\theta+r\sin\theta\tan\phi_{1}}{[XZ]}=\frac{r(\cos\theta+\sin\theta\tan\phi_{1})}{[XZ]}$ (S3)

and

$\frac{[OB]}{[YZ]}=\frac{\left[ ON \right]+[BN]}{[YZ]}=\frac{r\cos\theta+r\sin\theta\tan\phi_{2}}{[YZ]}=\frac{r(\cos\theta+\sin\theta\tan\phi_{2})}{[YZ]}$ (S4)

From triangle similarity, we have $\frac{[OA]}{[XZ]}=\frac{[OB]}{[YZ]}$

Equating (S3) and (S4), we obtain

$\frac{r(\cos\theta+\sin\theta\tan\phi_{1})}{[XZ]}=\frac{r(\cos\theta+\sin\theta\tan\phi_{2})}{[YZ]}$ (S5)

or

$\frac{[XZ]}{[YZ]}\boldsymbol{=}\frac{\cos\theta+\sin\theta\tan\phi_{1}}{\cos\theta+\sin\theta\tan\phi_{2}}=\frac{1+\tan\theta\tan\phi_{1}}{1+\tan\theta\tan\phi_{2}}$ (S6)

where [XZ] and [YZ] are known (see above), and φ1 and φ2 have been determined from (S1) and (S2) above.

Denoting the ratio $\frac{\left[ XZ \right]}{\left[ YZ \right]}$ by Q (which is now known), we can solve for $\tan\theta$ from (S6) to obtain

$\tan\theta= \frac{Q-1}{\tan\phi_{1}-Q\tan\phi_{2}}$ (S7)

Since φ1 and φ2 are known from (S1) and (S2), (S7) can be used to calculate *tanθ*, and hence θ.

***Stage 2: Calculation of bird height (h)***

Referring to Figure S9, since triangles PAB and PXY are similar, we can write

$\frac{H-h}{H}= \frac{\left[ OA \right]+[OB]}{[XY]} = \frac{r[2\cos\theta+\sin\theta(tan \phi_{1}+\tan\phi_{2})]}{[XY]}$ (S8)

H and *r* are known, and φ1, φ2 and θ have been calculated from (S1), (S2) and (S7), respectively.

Hence, we can solve for h from (S8) to obtain

$h=H\left[ 1-\frac{r[2\cos\theta+\sin\theta(tan \phi_{1}+\tan\phi_{2})]}{[XY]} \right]$ (S9)

We note that φ2 will be positive or negative according whether Y is to the left or the right of G. (PG is the perpendicular dropped from P to the line XY, or its extension). If [XG] > [XY], X and Y are on the same side of G. If [XG] < [XY], X and Y are on opposite sides of G. In our example the negative value of φ2 is used when solving for θ in (S7), and for *h* in (S9).

Once the height of the thorax point (*h*) is known, the 3D coordinates of the thorax point and the head can be calculated using the grid interpolation procedure described in Section 2.1 of the main text.

***Stage 3: Calculation of roll angle (α)***

Referring to Figure S8 we note that U, the point on the floor directly beneath the camera, is the origin of the 3D co-ordinate system, with co-ordinates (0,0,0). P, the nodal point of the camera lens, has co-ordinates (0,0,H). We also know the 3D co-ordinates of X, Y and Z, which have been projected from the camera image to the floor. (As these points are on the floor, they will each have a *z* coordinate of zero). Using this information we calculate the 3D coordinates of A, which we denote by the 3D vector $\bar{A}$, as follows:

$\bar{A}=\bar{\mathrm{UP}}+\bar{\mathrm{PA}}$ (S10)

From triangle similarity, we have $\bar{\mathrm{PA}}=\bar{\mathrm{PX}} \left[ \frac{(H-h)}{H} \right]$. Hence (S10) can be expressed as

$\bar{A}=\bar{\mathrm{UP}}+\bar{\mathrm{PX}} \left[ \frac{(H-h)}{H} \right]$ (S11)

Similarly, the 3D coordinates of B and O are expressed by the vectors

$\bar{B}=\bar{\mathrm{UP}}+\bar{\mathrm{PY}} \left[ \frac{(H-h)}{H} \right]$ (S12)

and

$\bar{O}=\bar{\mathrm{UP}}+\bar{\mathrm{PZ}} \left[ \frac{(H-h)}{H} \right]$ (S13)

Next, we compute the 3D vectors $\bar{\mathrm{OA}}$ and $\bar{\mathrm{OP}}$ as

$\bar{\mathrm{OA}}=\bar{A}- \bar{O}$, and $\bar{\mathrm{OP}}=\bar{P}- \bar{O}$ (S14)

We can then use the unit vectors ${\bar{\mathrm{OA}}}_{u}$and ${\bar{\mathrm{OP}}}_{u}$, representing the directions of vectors $\bar{\mathrm{OA}}$ and $\bar{\mathrm{OP}}$, to compute the angle AOP, which we denote by γ ( Figure S8):

$\gamma=\cos^{-1} \left( {\bar{\mathrm{OA}}}_{u}.{\bar{\mathrm{OP}}}_{u} \right)$ (S15)

where (.) denotes the dot product.

From the angles AOR (θ) and AOP (γ), calculated from (7) and (15), we can calculate the angle ROP, denoted by ψ in Figures S8 and S9, as

$\psi=\gamma-\theta$ (S16)

We can also use the unit vectors ${\bar{\mathrm{OA}}}_{u}$and ${\bar{\mathrm{OP}}}_{u}$ to determine the unit vector $\bar{n}$, defining the normal to the plane POA (see Figure S8), as

$\bar{n}= \frac{\left( {\bar{\mathrm{OA}}}_{u}\times{\bar{\mathrm{OP}}}_{u} \right)}{\sin\gamma}$ (S17)

where $(\times)$ denotes the cross product.

To determine the roll angle of the bird, we need to determine the direction of the 3D vector $\bar{\mathrm{OR}}$ (Figure S7), which is the vector representing the direction of the right wingtip. We denote the unit vector in this direction by ${\bar{\mathrm{OR}}}_{u}$. This unit vector can be determined from its relationship to the orientation of three other known vectors, as follows. First, since angle(AOR) = θ, we may write

${\bar{\mathrm{OA}}}_{u}.{\bar{\mathrm{OR}}}_{u}= \cos\theta$ (S18)

Second, since angle(ROP) = ψ, we may write

${\bar{\mathrm{OP}}}_{u}.{\bar{\mathrm{OR}}}_{u}= \cos\psi$ (S19)

Finally, since $\bar{\mathrm{OR}}$ lies in the plane AOP, which is normal to $\bar{n}$, we may write

$\bar{n}.{\bar{\mathrm{OR}}}_{u}= 0$ (S20)

The 3D unit vectors ${\bar{\mathrm{OR}}}_{u}$, ${\bar{\mathrm{OA}}}_{u} ,$ ${\bar{\mathrm{OP}}}_{u}$ and $\bar{n}$ can be represented as

$\left[ \mathrm{OR}_{\mathrm{ux}} \mathrm{OR}_{\mathrm{uy}} \mathrm{OR}_{\mathrm{uz}} \right]$, $\left[ \mathrm{OA}_{\mathrm{ux}} \mathrm{OA}_{\mathrm{uy}} \mathrm{OA}_{\mathrm{uz}} \right]$, $\left[ \mathrm{OP}_{\mathrm{ux}} \mathrm{OP}_{\mathrm{uy}} \mathrm{OP}_{\mathrm{uz}} \right]$, and $\left[ n_{x} n_{y} n_{z} \right]$ where the suffixes *x*, *y* and *z* denote the *x*, *y* and *z* components of these vectors.

Equations (S18-S20) can thus be re-expressed as

$\mathrm{OA}_{\mathrm{ux}}\mathrm{OR}_{\mathrm{ux}}+\mathrm{OA}_{\mathrm{uy}}\mathrm{OR}_{\mathrm{uy}}+\mathrm{OA}_{\mathrm{uz}}\mathrm{OR}_{\mathrm{uz}}=\cos\theta$ (S21)

$\mathrm{OP}_{\mathrm{ux}}\mathrm{OR}_{\mathrm{ux}}+\mathrm{OP}_{\mathrm{uy}}\mathrm{OR}_{\mathrm{uy}}+\mathrm{OP}_{\mathrm{uz}}\mathrm{OR}_{\mathrm{uz}}=\cos\psi$ (S22)

and

$n_{x}\mathrm{OR}_{\mathrm{ux}}+n_{y}\mathrm{OR}_{\mathrm{uy}}+n_{z}\mathrm{OR}_{\mathrm{uz}}=0$ (S23)

Equations (S21-S23) can be written in matrix form as

$P*\mathrm{OR}_{u}^{'}=Q$ (S24)

where (*) denotes matrix multiplication

and where

$P=\left[ \begin{matrix} \mathrm{OA}_{\mathrm{ux}} & \mathrm{OA}_{\mathrm{uy}} & \mathrm{OA}_{\mathrm{uz}} \\ \mathrm{OP}_{\mathrm{ux}} & \mathrm{OP}_{\mathrm{uy}} & \mathrm{OP}_{\mathrm{uz}} \\ n_{x} & n_{y} & n_{z} \end{matrix} \right]$, $Q=\left[ \begin{matrix} \cos\theta\\ \cos\psi\\ 0 \end{matrix} \right]$, and ${OR'}_{u}=\left[ \begin{matrix} \mathrm{OR}_{\mathrm{ux}} \\ \mathrm{OR}_{\mathrm{uy}} \\ \mathrm{OR}_{\mathrm{uz}} \end{matrix} \right]$ is the transpose of $\mathrm{OR}_{u}$ (S25)

Since the elements of P and Q are known, $\mathrm{OR}_{u}^{'}$ (and hence $\mathrm{OR}_{u}$) can be computed from (S24) using the matrix inverse of P:

$\mathrm{OR}_{u}^{'}=P^{-1}*Q$ (S26)

$\bar{\mathrm{OR}_{u}}$ is the unit vector that defines the direction of $\bar{\mathrm{OR}}$, which is the 3D direction of the right wing when it is fully extended. The roll angle α is the angle between $\bar{\mathrm{OR}_{u}}$ and the horizontal reference plane (see Figure S8). To compute this angle, we first calculate the angle β between $\bar{\mathrm{OR}_{u}}$ and the vertical (*z*) direction (not shown in Figure S8). Denoting the vertical direction by the unit vector $\bar{v}=\left[ \begin{matrix} 0 & 0 & 1 \end{matrix} \right],$β can be calculated from

$\beta=\cos^{-1} \left( \bar{v}. \bar{\mathrm{OR}_{u}} \right)$ (S27)

The roll angle α is then given by

$\alpha={90}^{\circ}-\beta$ (S28)

In our formulation the roll angle α is positive or negative according to whether the right wingtip is higher or lower than the left wingtip.

**SECTION D**

**Reconstruction of 3D trajectories when the wingspan is unknown**

Here we demonstrate that, even when the wingspan of the bird is not known, the 3D flight trajectory can be reconstructed in units of wingspan, regardless of whether or not the bird is rolling.

***Zero-roll condition:***

Referring to the main text, equations (2), (3) and (6) can be used to express (8) as:

$\left( xc,yc,zc \right)=\left[ \left( \frac{\omega}{W} \right)\frac{\left( XL+XR \right)}{2},\left( \frac{\omega}{W} \right)\frac{\left( YL+YR \right)}{2},H\left( 1-\frac{\omega}{W} \right) \right]$ (S29)

Denoting the depth of the bird below the ceiling by $h'$, we have $h^{'}=H-zc=\left( \frac{\omega}{W} \right)H$.

Expressing the 3D co-ordinates of the center of the bird in terms of $\left( xc,yc,h' \right)$, we can re-express (S29) as

$\left( xc,yc,h' \right)=\left[ \left( \frac{\omega}{W} \right)\frac{\left( XL+XR \right)}{2},\left( \frac{\omega}{W} \right)\frac{\left( YL+YR \right)}{2},\left( \frac{\omega}{W} \right)H \right]$ (S30)

Thus, the 3D position of the bird (and its trajectory and velocity) can be specified in terms of the wingspan unit (ω), which acts as a scale factor.

***Non-zero roll angle:***

If the roll angle is not zero, the angles θ and α can continue to be evaluated even when the wingspan is unknown, as is evident from equations (S7) and (S10 - S27). The depth (*h’*) of the thorax of the rolling bird, expressed in wingspan units (ω), can then be computed from equation (S9) as

$h^{'}=H-h=\left( \frac{\omega}{2ℇ} \right)H$ (S31)

where $ℇ=\frac{[XY]}{[2\cos\theta+\sin\theta(tan \phi_{1}+\tan\phi_{2})]}= \frac{W}{[2\cos\theta+\sin\theta(tan \phi_{1}+\tan\phi_{2})]}$ (S32)

(When the roll angle θ is zero, $ℇ=\frac{W}{2}$ and (S31) reduces to $h^{'}=\left( \frac{\omega}{2ℇ} \right)H$, which coincides with the result shown in (S30)).

Once the height *h* of the thorax above the floor $\left[ h=(H-h^{'}) \right]$ is known, the 3D coordinates of the thorax and the head can be evaluated (in wingspan units) using the grid interpolation procedure described in Section 2.1 of the main text:

$$\left( xc,yc,zc \right)=\left[ \frac{XC}{Q},\frac{YC}{Q},h \right]$$

and

$$\left( xH,yH,zH \right)=\left[ \frac{XH}{Q},\frac{YH}{Q},h \right]$$

Thus, even if the wingspan of the bird is not known, it is possible to obtain several scale-invariant properties of the bird’s trajectory such as its shape, tortuosity, slope of ascent/descent and roll angle, as well as the timing and features of salient temporal events such as the onset of accelerations or decelerations, or the frequency of oscillatory movements.
