# Supplementary material for: 3D reconstruction of bird flight trajectories using a single video camera
Source: PLoS One. 2022 Aug 24;17(8):e0271618. doi: 10.1371/journal.pone.0271618 (PMC9401184; doi:10.1371/journal.pone.0271618)
Supplement: Attachment — Submitted filename: Response to Reviewers.pdf [file pone.0271618.s005.pdf]

RESPONSE TO REVIEWERS

We thank the reviewers for their thorough examination of the manuscript, and for their valuable comments and suggestions for improvement.

Our responses to the comments and suggestions are listed below, point by point, in blue.

Reviewer 1

Major comments:

When reading the abstract of this submission I hoped to read about an approach that would enable 3D analysis of the multitude of single-camera bird flight videos uploaded to social media worldwide. That was not to be, but I did find the extended exploration of the method from (Vo et al 2017) and its extension to roll mathematically well executed and carefully explained. However, I believe the assumptions and requirements of the method limit its impact and applicability.

Thank you for this appraisal of the study.
We agree that it would be wonderful if the approach could be used for the reconstruction of 3D flight trajectories of flying birds from videos posted on social media. But this expectation is very unrealistic, as these posts rarely carry information about the calibration parameters of the video camera, or even the angular extent of its visual field - let alone information on lens distortions, etc. However, we believe that our technique can deliver useful information in those instances which meet the requirements that are set out in the manuscript, and which are discussed further below in response to the questions and comments.

For example, the procedure requires that the birds being measured flap regularly with maximally extended wings. Species as varied as pigeons, swallows, and seagulls commonly violate this ideal, especially during natural flight in the field.

The technique that we have developed does not require the wings to flap at a regular frequency. The height of the bird is determined each time the wings are outstretched, and this does not have to occur at regular intervals. The heights at intervening video frames are computed through linear interpolation. And when the bird glides with its wings stretched, the height can be computed at every frame. Incidentally, we find that, at least in the conditions of our experiments, the Budgerigars display a fairly regular wingbeat frequency (as in Fig. 4), except for occasional flap-bound maneuvers.

The extension to measuring roll angle assumes that at mid-downstroke the wings will be separated by a 180 degree angle, but this is also not necessarily the case for birds engaged in flapping maneuvers.

This really depends on how one defines the roll angle. If we consider the roll angle to be the roll angle of the head (or neck), this can be measured only with a frontal-view camera. Besides, it is questionable whether head roll represents the true roll angle, because many birds are known to partially or fully compensate for body roll by keeping the head horizontal. If the roll angle is taken to be the roll angle of the thorax, we agree that this angle may not necessarily coincide with the angle of the line connecting the wingtips at the time of maximum wing extension. However, it is very difficult to determine the roll angle of the thorax, even with high-resolution stereo cameras. We have therefore defined the bird's roll angle as the roll angle of the line connecting the wingtips when the wings are fully extended, i.e. separated by a 180 degree angle. This full wing extension must surely occur at some point during the wing downstroke - unless the downstroke is aborted prematurely, which is a rare occurrence. We also note that the roll angle that we infer (as per our definition of this angle) is only an incidental by-product of our analysis for reconstruction of the 3D flight trajectory. It is an intermediate step in the calculations and does not affect the accuracy of the 3D reconstruction - even if this angle does not accurately represent the roll angle of the thorax (for example). We have clarified this by adding a point (point vi) in the Discussion on p. 23.

Finally, the claim that the system could easily be _deployed_ in the field might be true, but the manuscript contains no evidence that it could easily be applied to field measurement.

The present study is a first step in which we have developed and validated a technique for reconstructing the 3D flight trajectory of a bird under laboratory conditions. The next step – examining the accuracy of this technique for tracking birds in natural outdoor conditions – would be the subject of a subsequent study. The accuracy of the method would deteriorate for birds with small wingspans or birds at a large distance from the camera, because the technique relies on a precise measurement of the distance between the thorax point and the extended wingtips in the camera image. This can be ameliorated to some extent by using a camera with higher pixel resolution. We have now included these considerations in the Discussion (new point vii, p. 24).

Using a species mean wingspan for measurement of birds recorded in the field is far more problematic than suggested by the authors because the physical properties of individual bird become mathematically correlated with their measured positions. Consider, for instance, a V formation of birds of the same species but slightly varying size. Birds with different actual wingspans will have different measured speeds, despite all the animals flying at the same speed together in formation. Similar problems occur even for recordings of isolated individuals, since other common quantities of interest such as flapping frequency are expected to co-vary with body size and speed, but in this method speed ends up correlated with the unmeasurable actual size of the animals. These issues appear to limit the wider impact of the method, though it may become a useful tool for some types of lab measurements.

Our technique does indeed rely on knowledge of the bird's wingspan for accurate reconstruction of its 3D flight trajectory. However – as we have already pointed out in the Discussion [item i), p. 24, 'Potential future applications…']– the method can deliver useful flight information even if the wingspan is unknown; for example, the flight speed (scaled to wingspan lengths per unit time), the exact 3D shape of the flight trajectory (scaled to wingspan length), its tortuosity, the slope of ascent/descent, the wingbeat frequency and other salient temporal events such as the onset of accelerations or decelerations, the frequency of oscillatory movements and the duration of intermittent gliding phases.

We agree with the Reviewer that difficulties can arise with reconstruction of the 3D tracks of birds in a flock if their individual wingspans differ significantly, even if they are all of the same species. Thank you for pointing this out. If the heights of all the birds are approximately the same over a short period of time (or vary only by a small percentage), then one way to deal with this wingspan variability would be to (i) measure the image wingspans (image distances between the extended wingtips) for each bird over this time period; (ii) compute the mean image wingspan across all the individuals in the image; (iii) select the individual with the image wingspan that is closest to the mean image wingspan to be the bird whose physical wingspan matches the known average physical wing span for the species; (iv) estimate the physical wing span of each bird through appropriate scaling based on its image wingspan. Once the physical wingspan of each bird has been estimated in this way, the usual technique can be applied to reconstruct the 3D trajectory of each individual in the flock. As mentioned above, this procedure relies on the assumption that the heights of the birds are approximately the same (or vary by only a small percentage) over the short period of time during which their image wingspans are measured. This assumption may not be valid for a flock that flies at a low altitude (because the percentage variation in altitude across individuals could be large), but it is likely to be quite accurate for flocks flying at high altitudes. This is because the percentage variation in altitude across the individuals would then be small compared to the percentage variation in their wingspans, so that variations in the image wingspans across the birds would then be almost entirely due to variations in their physical wingspans, rather than to variations in altitude. This could be one situation where the (unknown) variability in wingspans across individuals of a bird species can be circumvented by taking advantage of a large sample size. We have included these considerations in item ii) of the discussion of potential future applications on p. 24.

Variations in wing flapping frequency *per se* should not affect the accuracy of the technique, as long as the wingspan is known. A lower flapping frequency would simply lead to a longer period between successive wing extensions, and hence a longer interpolation of the trajectory between the extensions.

Minor comments:

Some of the extended supporting evidence for the base case would be better moved to an appendix. Figures 7 and 8 in particular add little to a manuscript where figures 6 and 9 are also present.

Thank you for this suggestion. We would prefer to leave Figure 7 in the main text, as it depicts the behavior during flight through an aperture that is narrower than the wingspan, which we compare with the behavior during flight through an aperture that is wider than the wingspan (Figure 6). However, we have moved Figure 8 to Section A of the Supplementary Information.

Instead of focusing solely on roll, section 3.2 should be generalized to all cases the line connecting the tips at mid downstroke is not in a plane perpendicular to the camera optical axis. Roll is one such case, but so is a bird in level flight viewed by a camera with an optical axis not perpendicular to gravity. Relaxing constraints on camera placement makes the method applicable to a wider range of lab and especially field recording situations.

Thank you for raising this important point. It turns out that the general method (described in Section B of the Supplementary information) will work even when the camera's optical axis is not perpendicular to gravity. Regardless of the orientation of the camera's optical axis, the method will deliver the bird's 3D position and roll orientation *relative to the plane of the calibration grid*, which is perpendicular to the camera's optical axis. The bird's 3D position and roll angle, computed relative to this plane, can then be transformed to the horizontal plane using 3D transformational geometry, from the known orientation of the camera's optical axis relative to the horizontal plane. We have clarified this in a new point (point ix) added to the Discussion (p. 22).

"Examples of images acquired by the overhead camera." Are these the full-frame test images? The bird appears to nearly fill the field of view of the camera, an implausible configuration for most lab experiments and quite different than Figure 4.

These are full-frame images. The demonstration could have been performed with a smaller model bird (or a larger grid), but the aim of this exercise was simply to demonstrate that the general procedure for computing 3D height delivers the correct, ground-truth values even when the bird is rolling and the thorax point is not directly below the camera (i.e. even when it is not in line with camera's optical axis). The precision of the method depends upon the image resolution of the camera, and would deteriorate when the bird is at a large distance from the camera. We have now specifically noted this limitation in a new point (point vii) added to the Discussion (p. 24).

It would be interesting to see how the uncertainty in this method fares, especially for distant objects represented by few pixels such as birds observed in the field. Stereo methods are limited in part by the length of the stereo baseline, but while this method doesn't have a stereo baseline per se it seems like the bird's wingspan is the equivalent length information and might be problematically small for objects more than a few meters away. The roll-safe method seems to compound the problem by using half-span distances.

As we mention in the Discussion (new point vii, p. 24), the accuracy of the results would deteriorate for birds with smaller wingspans or birds at a large distance from the camera, because it relies on precise measurements of the distances between the thorax point and each extended wingtip in the camera image. This issue can be ameliorated to some extent by using a camera with higher pixel resolution.

Advantage iv), that the calibration grid can be removed after calibration does not appear to be an advantage at all, since no comparable method requires a grid in the first place.

All stereo-based methods require the use of a calibration grid (or some other standard pattern of markers) to determine (a) the image distortions produced by the lens of each camera; (b) the separation between the nodal points of the two cameras (the stereo baseline); and (c) the relative orientations of the two camera axes. Typically, this is achieved by using multiple images of a moving calibration checkerboard, captured simultaneously by both cameras (e.g. the Jean-Yves Bouguet stereo calibration technique).

Our method of calibration is far simpler - it only requires a single snapshot of a calibration grid with its plane oriented perpendicular to the camera's optical axis. This grid does not need to be painted on the floor. It can be positioned at any distance from the camera (even closer than the bird), and can be printed as a checkerboard. The calibration grid can be superimposed on the bird video images when the thorax point and extended wingtip positions are being digitized. Once the snapshot of the calibration grid is acquired, the camera's imaging properties (including all distortions) are fully registered and there is no need for further calibration, regardless of where the camera is deployed.

Calibration of optical distortion with the grid method proposed here seems unnecessarily difficult. At minimum you should use a checkerboard grid, so the widely available and easy-to-use checkerboard corner detection algorithms can be used. Better still, the same information can be pulled out of a pinhole camera lens distortion model, allowing use of the highly automated procedures that exist for measuring those parameters. You mention that the grid method can deal with unusual distortion cases and that's true, but such are rarely encountered and the most common ones, extreme fisheye lenses, would also be difficult to calibrate with the grid method.

The calibration grid laid out on the floor is, in effect, a checkerboard. The intersection points of the grid can be selected manually or detected automatically using simple image-processing techniques, if one wishes. This is not a tedious task even if it is performed manually, as the calibration procedure requires the use of just a single image of the calibration pattern – for the entire lifetime of the camera. The calibration pattern that we have used for validating the extended procedure is, in fact, a checkerboard (Figs 12, 13). As we have pointed out on p. 23 in item iii) of the Discussion, the calibration pattern does not need to be on the floor. It can be in a parallel plane that is much closer to the camera than the bird – for example, a few centimetres away, to facilitate the calibration of large visual fields. This calibration grid can be superimposed on the bird video images when the thorax point and extended wingtip positions are being digitized. A camera carrying a fish-eye lens (with a nearly hemispherical field of view) could be calibrated by creating a spherical calibration grid (e.g. lines of latitude and longitude) on a transparent hemispherical shell affixed to the front of the camera, with its centre located at the nodal point of the camera's lens. We have now noted (following the Reviewer's suggestion) that one could also use algorithms that are available for calibrating optical distortions in pinhole cameras (e.g. the Jean-Yves Bouguet algorithm), provided they are able to capture and characterize all of the relevant optical distortions accurately. We have added these considerations in item iii) on p. 23.

Advantage vii) that this method is computationally simple – sure, but so are the triangulation calculations of stereo methods. Even the least Raspberry Pi has more than adequate compute performance for these tasks. It's localizing the animal in the image that is the hard part, and that's broadly similar in both cases, perhaps even more challenging with the method here since two pixel locations are needed from an image instead of one.

We agree that stereo-based triangulation methods are also simple, but our method uses a single camera, whereas a stereo method requires at least two cameras. Furthermore, a stereo system would have to be recalibrated each time it is moved to a new location, because the baseline and the relative orientations of the two cameras are likely to change - unless the two cameras are carried by a large, rigid supporting framework, which can be impractical in the field.

Reviewer 2

Major comments:

Srinivasan, Vo, and Schiffner provide a thorough description of a simple and low cost technique for tracking the 3D trajectory of flying birds. This is a highly relevant contribution because there are a growing number of investigators, including many junior investigators, that are interested in avian flight but working outside of the handful of labs with the currently available methods. The new method is clearly presented and validated in this manuscript. It has also been used successfully in several recent and important studies in avian visual guidance. Thus, there is abundant evidence that the method works and produces useful data. The manuscript is also very well written so the method really will be fully available to any smart person willing to give it a try.

Thank you for these useful and positive comments.

My only significant advice is that the cases in which a bird in rolling and not directly underneath the camera are probably going to be the majority of cases. Thus, I would encourage the authors to extend section 3.2 to provide the same high-level description of these cases. This change may lead to a doubling of the size of that section or the insertion of a new section 3.3. However, given how common this phenomenon will be, I would encourage the authors to include more about it in the main text.

This comment appears to have arisen from a misunderstanding of our approach to include the effects of roll, which is actually quite general. In Section 3.2 we introduce the principle behind the approach by considering a simple example in which the thorax of the bird is directly beneath the camera. In the Supplementary Information (Section B) we derive an extension of this calculation for the general case, in which the bird can be at any 3D location. The extended calculation delivers the height of the bird, as well as the roll angle, irrespective of the position of the bird relative to the camera. We had pointed this out in the final paragraph of Section 3.2, but we have now modified the wording of this paragraph, as well as the wording of the second paragraph in Section 3.2, to improve clarity. We have added further clarification by including two additional sentences in the legend to Figure 13: "Note that it is not necessary for the thorax point to be directly below the camera (see, for example, (b) and (d)). The optical axis of the camera intersects the grid plane at its center."

At the time of drafting the manuscript, we did consider incorporating Section B of the Supplementary Information into the text of the main manuscript (immediately following Section 3.2). However, we did not pursue this as we felt that it would make the main manuscript unduly lengthy.

Major comments:

ms page 2: "attached to the tracked" is something missing here?

Thank you for spotting this typographical error, which has now been corrected.

fig 1 caption (and throughout): why is "i.e." written without a comma after it? Isn't that standard punctuation? Please correct me if I'm wrong. I think of the lead author as a master of the English language (in regards to science writing) so please let me know if I'm mistaken.

We believe that both forms are commonly used, but we have adopted the Reviewer's advice and included a comma after "i.e." throughout the manuscript.

ms page 4: shouldn't "this data is used" be "these data are used"?

Thank you for spotting this grammatical error, which has now been corrected.

ms page 10: what is yjorax?

Thank you for spotting this typographical error, which has now been corrected.

ms page 13 (and throughout): I suggest using "wingbeat" instead of "wing beat"

Thank you for this suggestion, which has now been incorporated throughout the manuscript.

****
