# Supplementary material for: 3D reconstruction of bird flight trajectories using a single video camera
Source: PLoS One. 2022 Aug 24;17(8):e0271618. doi: 10.1371/journal.pone.0271618 (PMC9401184; doi:10.1371/journal.pone.0271618)
Supplement: Attachment — Submitted filename: Srinivasan_et al_response_to_reviewers.pdf [file pone.0271618.s006.pdf]

Responses to review

Dear Editor,

We are glad to hear that the manuscript is potentially acceptable for publication, subject to satisfactory minor revisions. Our responses to the reviewer's suggestions are outlined below (in blue).

1. More details on the spacing and resolution of the calibrating grid may be of help to others attempting to replicate the method (lines 73-74). Whilst more information about the grid used by the authors to calibrate their system is given later in the manuscript (lines 256-257), readers can benefit from a short discussion on any potential effect of using grids of different dimensions to those presented by the authors. More precisely, is there any potential effect on the calibration if using a grid of dimensions different to those used by the authors? Is there a minimum grid size required to attain an accurate calibration?

Thank you for raising this point. While there are benefits to increasing the grid density, there are also some drawbacks. We now discuss the tradeoffs briefly in lines 118-126.

2. I think that labels for the y-axis of Figure 8 needs some editing. As the important information here is the position of the thorax and head of the bird (relative to the ground?) for the different slits tested, perhaps the y-axis may use a different label and units.

We apologize for the confusion that this labeling may have caused. The term "Tunnel width" does not refer to the width of the tunnel, but, rather, to the position (in meters) along the width of the tunnel (corresponding to the *y* axis in Fig. 3). This notation is used consistently in figures (5-8), and (S1-S6). We have clarified the meaning of the notation by adding an explanation in the legend of Fig. 5, where it is first used (lines 296-298).

Please note that the line numbers cited here for the revisions refer to the text in the revised manuscript *per se*. They may differ slightly from the line numbers in the version that is compiled by the submission website.

Revisions have also been made in the SI, which now includes a new Section (Section A) that provides the additional information required by the ARRIVE 2.0 Guidelines checklist.

On behalf of all the authors,

Mandyam Srinivasan
Corresponding author
